# Tip detection-antegrade dissection and re-entry (TD-ADR) with integrated fluoroscopic and intravascular ultrasound images in chronic total occlusion: first case report of integrated TD-ADR technique

**DOI:** 10.1093/ehjcr/ytae378

**Published:** 2024-08-14

**Authors:** Yutaka Tadano, Shoichi Kuramitsu, Takuro Sugie, Daitaro Kanno, Tsutomu Fujita

**Affiliations:** Department of Cardiovascular Medicine, Sapporo Cardio Vascular Clinic, Sapporo Heart Center, North 49, East 16, 8-1, Higashi Ward, 007-0849, Sapporo, Japan; Department of Cardiovascular Medicine, Sapporo Cardio Vascular Clinic, Sapporo Heart Center, North 49, East 16, 8-1, Higashi Ward, 007-0849, Sapporo, Japan; Department of Cardiovascular Medicine, Sapporo Cardio Vascular Clinic, Sapporo Heart Center, North 49, East 16, 8-1, Higashi Ward, 007-0849, Sapporo, Japan; Department of Cardiovascular Medicine, Sapporo Cardio Vascular Clinic, Sapporo Heart Center, North 49, East 16, 8-1, Higashi Ward, 007-0849, Sapporo, Japan; Department of Cardiovascular Medicine, Sapporo Cardio Vascular Clinic, Sapporo Heart Center, North 49, East 16, 8-1, Higashi Ward, 007-0849, Sapporo, Japan

**Keywords:** Case report, Percutaneous coronary intervention, Coronary artery disease

## Abstract

**Background:**

Tip detection-antegrade dissection and re-entry (TD-ADR) technique allows operators to accurately observe both guidewire tip direction and a true lumen in chronic total occlusion (CTO) lesions, while the torque direction of the guidewire on IVUS images does not invariably correspond to that on fluoroscopic images.

**Case summary:**

A 41-year-old man with hypertension who smokes presented with sudden onset of dyspnoea, acute heart failure, and ischaemic findings on electrocardiogram; we performed percutaneous coronary intervention (PCI) for a sub-totally occluded mid-left anterior descending artery lesion. All antegrade wiring attempts failed to enter the distal true lumen followed by subintimal tracking and re-entry technique. Since the lesion re-occluded the next day, we treated the lesion using a novel TD-ADR technique, termed the ‘integrated TD-ADR’, because of no interventional retrograde channel. This method integrates fluoroscopic and intravascular ultrasound (IVUS) images, ensuring congruence in the torque direction of the guidewire across both modalities and enabling vertical puncture of the stiff guidewire from the extraplaque space to the distal true lumen quickly and precisely. Final angiography showed good results. Five months later, coronary angiography showed that the lesion remained open.

**Discussion:**

The integrated TD-ADR technique merges fluoroscopic and IVUS images, allowing operators to torque the guidewire in the same direction on both images. This approach might be more user-friendly than the original technique and has the potential to enhance the success rate of PCI in complex CTO cases. However, further investigations are warranted to address the clinical feasibility and applicability of this technique.

**Learning points**

- To know a refined technique of intravascular ultrasound-guided wire-based antegrade dissection and re-entry using the tip detection method.
- This method synchronizes the orientation of the ultrasound image with the fluoroscopic view, enabling more precise manipulation of the guidewire within chronic total occlusion lesions.

## Introduction

The success rate of percutaneous coronary intervention (PCI) for chronic total occlusion (CTO) has significantly improved, increasing from 50–70% to 85–94%; however, advanced techniques [e.g. retrograde approach, antegrade dissection and re-entry (ADR), and intravascular ultrasound (IVUS)-guided wiring] are required to achieve procedural success in challenging cases.^1^ Recently, Okamura *et al*. reported a tip detection-ADR (TD-ADR) technique using a new CTO-specific IVUS system (AnteOwl, Terumo, Japan).^2–4^ Although the TD-ADR does not require angiographic observation, discrepancies may occur in guidewire torque direction between the fluoroscopic and IVUS images. To address these limitations, we report a case of a novel TD-ADR method that integrates both fluoroscopic and IVUS images.

## Summary figure

**Figure:**
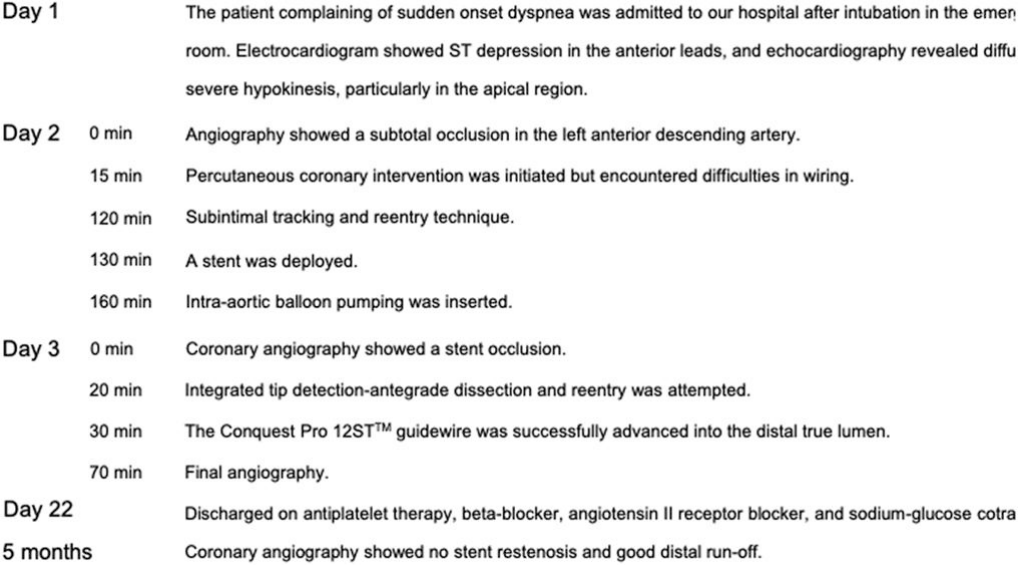


## Case presentation

**Figure 1:**
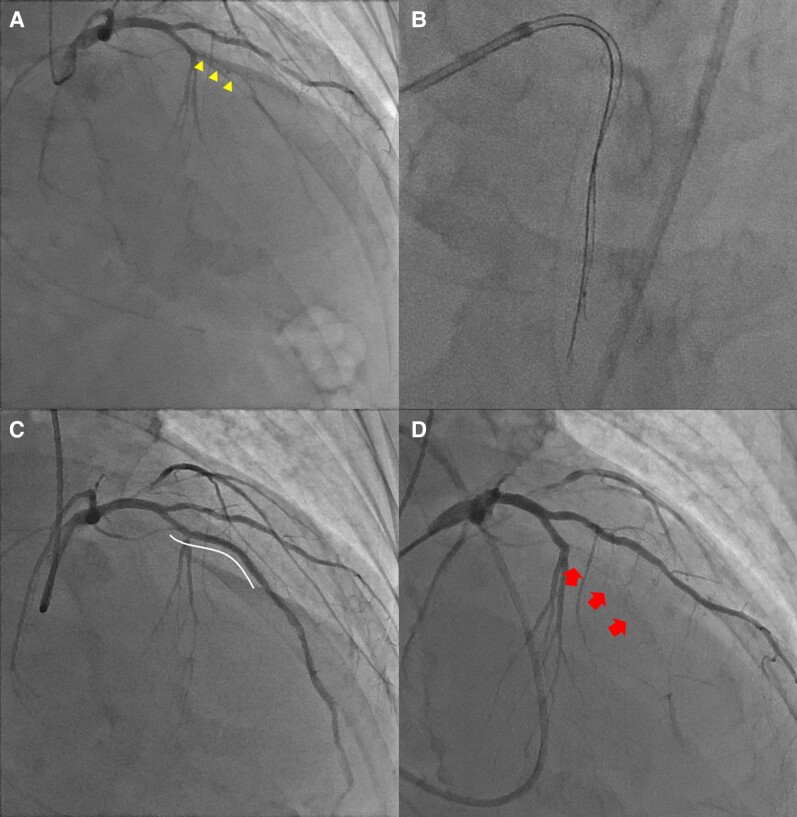
Emergent coronary angiography and intervention. (*A*) Emergent coronary angiography. (*B*) Conventional intravascular ultrasound (IVUS)-guided wiring. (*C*) Final coronary angiography at the index procedure. A 3.0 × 48 mm drug-eluting stent was implanted at the mid-left anterior descending coronary artery (white line). (*D*) Coronary angiography the next day after the index procedure.

**Figure 2:**
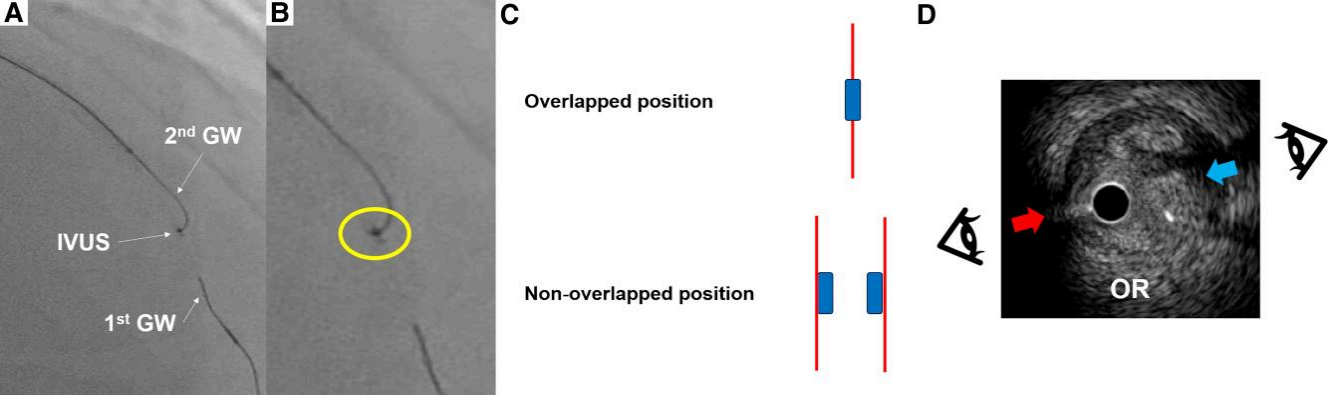
Identifying the fluoroscopic viewing direction based on the positional relationship between the first guidewire and IVUS catheter. (*A* and *B*) The first guidewire and intravascular ultrasound (IVUS) catheter appears overlapped from the right cranial fluoroscopic view. Also, the tip of the second guidewire looks like downward direction. (*C*) The relationship between the IVUS catheter and the first guidewire is classified into overlapped and non-overlapped positions based on the IVUS transducer (box) and the first guidewire (line). (*D*) The fluoroscopic viewing direction is from either 2 or 8 o’clock according to the positional relationship between the first guidewire and IVUS catheter.

**Figure 3:**
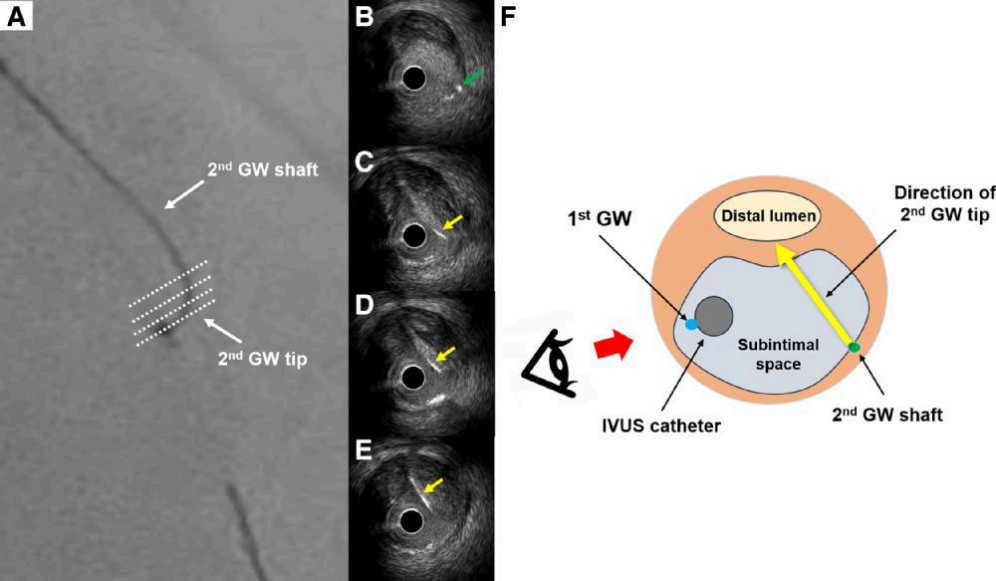
Confirming the fluoroscopic viewing direction based on the positional relationship between the IVUS catheter and second guidewire. (*A*) The second guidewire shaft is located on the right side of the intravascular ultrasound (IVUS) catheter, and its tip is towards the IVUS transducer. (*B–E*) Serial IVUS cross-sectional images from proximal to distal left descending coronary artery. (*F*) The fluoroscopic viewing direction on IVUS images is from 8 o’clock.

**Figure 4:**
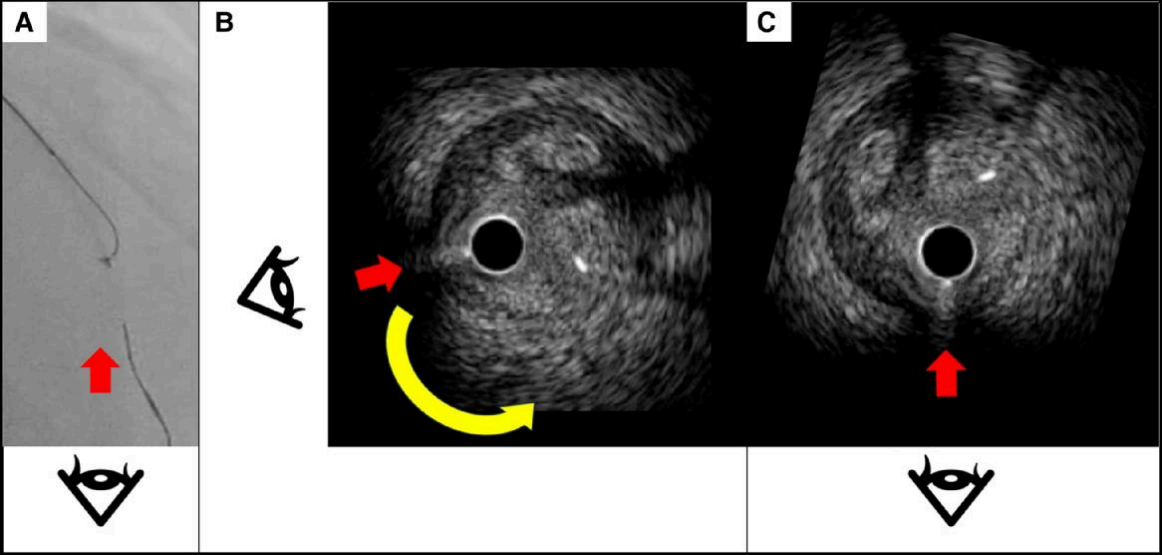
Integrating fluoroscopic and IVUS images to torque the guidewire in a same direction. (*A*) The fluoroscopic viewing direction is defined as 6 o’clock in mid-left anterior descending coronary lesions from the cranial fluoroscopic view. (*B* and *C*) To align the viewing direction on fluoroscopy and intravascular ultrasound (IVUS), IVUS images are rotated 70° counterclockwise. Thus, the downward direction on the fluoroscopic image corresponds to the direction from 2 to 10 o’clock on the IVUS image.

**Figure 5:**
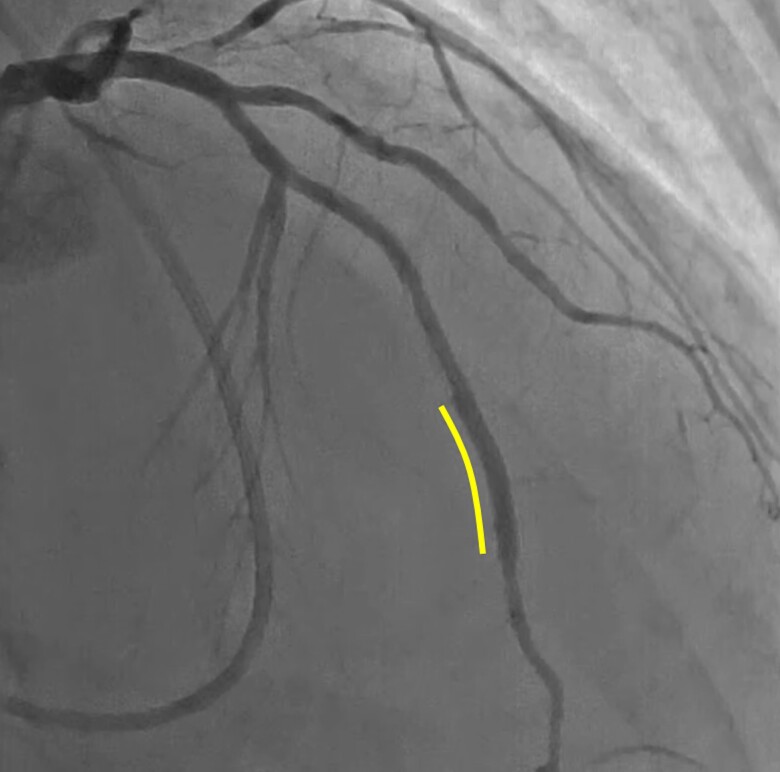
Final angiographic results. A 3.0 × 24 mm drug-eluting stent was implanted at distal left anterior descending coronary artery.

A 41-year-old man with a prior history of hypertension and current smoking was referred to our hospital due to sudden onset of dyspnoea. In the emergency room, his blood pressure was 97/61 mmHg, pulse 114/min, and oxygen saturation level 78%; he was immediately intubated. Electrocardiography showed ST-segment depression in the anterior leads. Echocardiography revealed reduced wall motion throughout the left ventricle (ejection fraction: 35%), particularly in the apical region. Blood tests showed a remarkably high level of brain natriuretic peptide (1688.1 pg/mL; normal: 0–18.4 pg/mL) with slightly elevated cardiac enzymes, including creatine kinase (300 U/L; normal: 59–248 U/L) and creatine kinase MB (26 U/L; normal: 0–25 U/L). Emergency coronary angiography was performed a day after admission, revealing a sub-totally occluded lesion in the mid-left anterior descending coronary artery (LAD) (*Figure 1A*). Antegrade wire escalation and IVUS-guided wiring were attempted but failed to enter the distal true lumen (*Figure 1B*). Finally, a distal re-entry was created using the subintimal tracking and re-entry technique (STAR), and a drug-eluting stent was implanted in the subintimal space (*Figure 1C*; Supplementary material online, *Video S1*). However, heart failure did not improve after the PCI. Coronary angiography revealed a re-occluded lesion in the mid-LAD (*Figure 1D*). We decided to treat the lesion again via an antegrade approach because there was no interventional retrograde channel. The procedure was performed with a 7 Fr guiding catheter via the radial artery. A SION Black (Asashi Intecc, Japan) with a microcatheter was advanced into the lesion and entered the subintimal space, as confirmed by AnteOwl IVUS. Since IVUS revealed that the inner lumen was maintained ∼5 mm beyond the proximal stent edge, we attempted TD-ADR using the Conquest Pro 12 Sharpened Tip (CP12ST). In the fluoroscopic view, the first guidewire (SION Black) and the IVUS transducer appeared overlapped (*Figure 2A–C*) based on the IVUS transducer and the first guidewire position, suggesting that the fluoroscopic viewing direction on the IVUS cross-sectional image is from either 2 or 8 o’clock (*Figure 2D*). Next, the second guidewire shaft was located on the right side of the IVUS catheter, with its tip directed towards the IVUS transducer (downward) (*Figure 3A*); serial IVUS images revealed that the second guidewire tip is directed from 4 to 12 o’clock (*Figure 3B–E*). Given these findings, the downward direction of the second guidewire’s tip on the fluoroscopic view corresponded to the 4–12 o’clock (upward) direction on the IVUS image. If the right anterior oblique cranial fluoroscopic direction was from 2 o’clock, the second guidewire’s shaft would be positioned on the left side of the IVUS catheter. Thus, it is deduced that the fluoroscopic viewing direction is from 8 o’clock (*Figure 3F*). On the other hand, we see the IVUS images in mid-LAD lesions on the cranial fluoroscopic view from 6 o’clock (*Figure 4A*). To align the viewing direction on fluoroscopy and IVUS, we rotated the IVUS images 70° counterclockwise (*Figure 4B* and *C*). Integrating fluoroscopic and IVUS images made manipulating the second guidewire more straightforward, enabling vertical sticking of the CP12ST into the distal true lumen (see Supplementary material online, *Videos S2*–*S4*). The SION Black passed through the lesion after advancing a microcatheter into the distal true lumen, as confirmed by IVUS (see Supplementary material online, *Videos S5*–*S7*). With stent implantation, final coronary angiography showed good results (*Figure 5*; Supplementary material online, *Video S8*). He was discharged on Day 22 with antiplatelet therapy, a beta blocker, an angiotensin II receptor blocker, and a sodium-glucose transport protein 2 inhibitor. Five months later, coronary angiography showed that the lesion remained open, although cardiac function was similar to that at discharge.

## Discussion

The TD-ADR technique allows for the real-time visualization of guidewire tip direction unlike conventional IVUS-guided wiring.^2–5^ Nevertheless, mastering the TD-ADR technique might be challenging for most interventionists owing to the need for extensive knowledge of IVUS and advanced skills.

In this case report, we introduce a novel TD-ADR technique integrating fluoroscopic and IVUS images to enhance usability. This new TD-ADR technique comprises three steps. Step 1 is to identify the fluoroscopic viewing direction where the IVUS transducer overlaps the first guidewire (*Figure 2*). Step 2 is to ascertain the precise fluoroscopic viewing direction according to the second guidewire’s tip direction and shaft location (*Figure 3*). Step 3 is to rotate the IVUS image to align with the fluoroscopic viewing direction (*Figure 4*). Regarding fluoroscopic viewing direction, we define 6 o’clock position as appropriate when manipulating a guidewire in mid-LAD lesions from cranial view. As operators need to be aware of three-dimensional wiring manipulation on fluoroscopic image when using the TD-ADR technique, synchronizing the viewing directions on fluoroscopy and IVUS images is crucial for imagining the distal true lumen on the fluoroscopic view, enabling more precise manipulation of the second guidewire.

To our knowledge, this case report is the first to propose a new TD-ADR technique, termed the integrated TD-ADR technique, which merges fluoroscopic and IVUS images. This technique enables operators to torque the guidewire consistently on both fluoroscopic and IVUS images, potentially increasing the success rate of CTO PCI. The AnteOwl IVUS has a shorter tip-to-transducer length (8 mm) than conventional IVUS catheters (e.g. OPTICROSS, Boston Scientific; 20 mm), allowing us to use this technique effortlessly. However, this new IVUS catheter is currently available only in Japan, reducing the generalizability of this technique. Conventional IVUS catheters can be applied when subintimal space is long enough to insert them.

Advanced techniques, including the retrograde approach and ADR, contribute to procedural success, especially in challenging CTO cases.^1,6^ However, these techniques are also associated with serious complications, such as coronary perforation.^7^ The current guideline has downgraded the recommendations for CTO PCI from class IIa to IIb.^8^ Given these facts, achieving both the safety and efficacy of CTO PCI is more crucial for operators than ever before. Recently, Tanaka *et al*.^4^ reported that the TD-ADR resulted in a higher success rate and shorter procedural time without complications compared to the Stingray-ADR, highlighting the clinical advantage of this approach. Since the integrated TD-ADR is more straightforward than the original, it has the potential to enhance the clinical applicability of TD-ADR in daily practice. However, further investigations are warranted to address the clinical feasibility and applicability of this technique.

## Conclusion

The integrated TD-ADR technique merges fluoroscopic and IVUS images, allowing operators to torque the guidewire in the same direction on both images. This approach is more user-friendly than the original technique and has the potential to enhance the success rate of PCI in complex CTO cases.

## Lead author biography


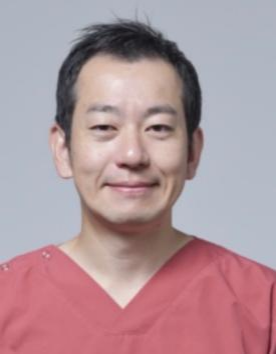


Yutaka Tadano was born in 1979 in Hokkaido, Japan. He earned his degree from Asahikawa Medical University in 2004. Currently, he serves as the Director of Cardiovascular Medicine at the Sapporo Cardio Vascular Clinic in Hokkaido, Japan. His primary expertise and interest lie in percutaneous coronary intervention.

## Supplementary Material

ytae378_Supplementary_Data

## Data Availability

The data underlying this article are available in the article and in its online Supplementary material.
