# Supplementary material for: Tip detection-antegrade dissection and re-entry (TD-ADR) with integrated fluoroscopic and intravascular ultrasound images in chronic total occlusion: first case report of integrated TD-ADR technique
Source: Eur Heart J Case Rep. 2024 Aug 14;8(8):ytae378. doi: 10.1093/ehjcr/ytae378 (PMC11354217; doi:10.1093/ehjcr/ytae378)
Supplement: ytae378_Supplementary_Data [file ytae378_supplementary_data.zip › Supplementary Materials.docx]

**Supplementary Materials**

**Supplementary Videos**

**Video S1. Final angiogram on the day 1.**

The blood flow of the left anterior descending artery is insufficient because the reentry site of the subintimal tracking and reentry technique is quite distal.
 **Video S2**. **Intravascular ultrasound during the integrated tip detection-antegrade dissection and reentry procedure.**

An attempt is being made to vertically stick with the Conquest Pro 12ST^TM^ guidewire.

**Video S3.** **Fluoroscopy during the integrated tip detection-antegrade dissection and reentry procedure.**

The Conquest Pro 12ST^TM^ guidewire is successfully penetrating the true lumen through to the septal branch.
 **Video S4**. **Intravascular ultrasound after the successful integrated tip detection-antegrade dissection and reentry.**

The Conquest Pro 12ST^TM^ guidewire is positioned inside the distal true lumen.

**Video S5. Fluoroscopy during the advancement of a microcatheter into the true lumen.**

The Finecross GT^TM^ microcatheter is being advanced along the Conquest Pro 12ST^TM^ guidewire under the real-time visualization using intravascular ultrasound. **Video S6**. **Fluoroscopy during wiring after guidewire swapping.**

After the microcatheter was advanced into the true lumen, the Conquest Pro 12ST^TM^ guidewire was swapped for the SION black^TM^ guidewire.

**Video S7**. **Intravascular ultrasound after the guidewire was advanced into the distal true lumen.**

The guidewire and intravascular ultrasound is positioned within the distal true lumen, surrounded by the expanded subintimal space.

**Video S8. Final angiogram on the day 2.**The blood flow of the left anterior descending artery has improved compared with that on the day 1.
